# 

**Published:** 1875-02

**Authors:** 


**TERMS, ALWAYS IN ADVANCE:** One copy, per annum.... $4 00

☞ Yearly subscriptions may begin with any number, Postage Free.

☞ Persons who send subscriptions for the JOURNAL will please consider the first number received after subscription as a sufficient receipt for money sent.

☞ We cannot return rejected manuscripts.

CHICAGO:

W. B. KEEN, COOKE & CO., PUBLISHERS,

NOS. 113 AND 115 STATE STREET.

Copyright, A.D. 1875. W. B. KEEN, COOKE & CO.

☞ Back Nos. for 1872, '73 and '74 can be supplied at 30 cents each.

S.G.O.L. INDEXED

CONTRIBUTORS TO

### The Chicago Medical Journal.

### JANUARY, 1875.

JOHN WOODBRIDGE, ESQ., Chicago.
M. A. McCLELLAND, M.D., Knoxville, Illinois.
JOHN R. CUSHING, M.D., Harrison County, Texas.
A. REEVES JACKSON, M.D., Chicago.
JNO. E. OWENS, M.D., Chicago.
R. E. STARKWEATHER, M.D., Chicago.
I. N. DANFORTH, M.D., Chicago.
F. C. WINSLOW, M.D., Chicago.
PROF. EDWIN POWELL, Chicago.
BEN. C. MILLER, M.D., Chicago.

### FEBRUARY, 1875.

M. A. McCLELLAND, M.D., Knoxville, Ill.
E. W. LEE, M.D., (Dublin), Chicago.
JNO. E. OWENS, M.D., Chicago.
PHILIP ADOLPHUS, M.D., Chicago.
J. L. WHITE, M.D., Bloomington, Ill.
E. L. HOLMES, M.D., Chicago.
PROF. MOSES GUNN, M.D., Chicago.
BEN. C. MILLER, M.D., Chicago.

